# Causal explanations within weak and incomplete theories

**DOI:** 10.3389/fpsyg.2015.01689

**Published:** 2015-11-03

**Authors:** Nikolai Axmacher

**Affiliations:** Department of Neuropsychology, Institute of Cognitive Neuroscience, Ruhr-University BochumBochum, Germany

**Keywords:** psychoanalysis, neuroscience, causality, causal explanations, neuropsychoanalysis, epistemology

In a previous article (Axmacher, 2013) I argued that neuroscientific and psychoanalytic explanations are in general epistemologically consistent with each other, even if psychoanalytic claims refer to (typically unconscious) reasons, whereas neuroscientific claims are about causes. I claimed that hermeneutic (psychoanalytic) explanations are not inconsistent with causal (neuroscientific) explanations even if they are typically given as “deferred reconstructions”–in other words, as *post-hoc* explanations of feelings, symptoms or behavioral patterns that initially appear irrational, random and senseless. Specifically, I claimed that psychoanalytic explanations—like (neuro)scientific explanations—are successful if and only if they can determine the sufficient conditions which give rise to the feeling, symptom, or behavioral pattern in question.

I was pleased to see that Dr. Elisa Galgut has critically discussed my argument from the point of view of Donald Davidson's anomalous monism (Galgut, 2014). She argues that there are no general psychological laws that are comparable to laws in the natural sciences: “a central difference between psychological explanations, and causal explanations in the sciences, is that the latter, but not the former, appeal to generalizable laws.”: “We can cite causes and effects of particular psychological events, but we can never cite the law under which these events are subsumed, because there are no psychological laws.” In particular, the list of possible boundary conditions on psychological laws is in principle infinite, so that there may always be exemptions from them. By contrast, according to her view this is not the case for causal explanations in the sciences, where the list of boundary conditions is limited. In propositional psychology “The set of disjunctives is open-ended as a matter of principle, whereas this is not the case in the physical sciences.”

I think that Davidson can be understood as arguing that physical laws are “stricter” than psychological laws because explanations in commonsense psychology—as well as in psychoanalysis—typically refer to propositional states such as desires, wishes, or conflicts (that result from several incompatible wishes). Propositional states are representations, which have several important properties: The link of representations to their contents is “looser” than the link between non-representational physical states or events (e.g., because changes in contents may not necessarily be accompanied by changes of the corresponding representations). Furthermore, representations are “holistic” in the sense that changes in one representation affect the entire network of representations.

I agree with Davidson (and Galgut) on these points. However, as we will see below, explanations in cellular neurosciences, cognitive neurosciences and psychoanalysis may also refer to representations, and therefore this is not a distinguishing property between neuroscientific and psychoanalytic explanations.

My argument is less philosophical than it is empirical: Even after excluding some outdated candidates, there are many theories about how causation and causal explanation should be conceived of (probably about as many as there are philosophers who discussed this topic). So, how do we know which one of the many current theories on this question is correct? I suggest to adopt a perspective similar to the one in the social sciences and to investigate which explanations are currently indeed used in neurosciences and in psychoanalysis.

As I have argued, psychoanalytical explanations (or, more generally, psychological explanations) share—if they are successful—the logical structure of neuroscientific explanations (as a specific kind of explanations in the physical sciences) in a specific respect. They are true if and only if the occurrence of an explanans (a cause or a reason) invariably leads to a specific effect: the explanans needs to be sufficient for the effect. (Notably, I do not believe that the truth of psychoanalytic or neuroscientific explanations requires that an explanans be *necessary* for an effect: In the absence of one specific cause or reason, other causes or reasons may lead to the same effect, and the explanation may still be valid. This implies over-determinism, which I agree weakens the explanatory power of neuroscientific or psychoanalytic explanations. But this is all we have—and explanations in terms of sufficient conditions are very important in all curative disciplines like medicine or psychotherapy, because they describe what needs to be done in order to reach a certain effect).

In both neuroscientific and psychoanalytic explanations related to representations, the relationship between an explanans and an effect is typically only true if a number of boundary conditions obtain as well. These conditions, as Galgut says, often cannot be spelled out—this again seriously weakens the explanatory power in the neurosciences and in psychoanalysis, but, again, this is all we have. If scientific laws related to representations are not based on an exhaustive list of conditions under which they are valid or invalid—if they only indicate some sufficient conditions which, under specific boundary conditions, lead to an effect—how are they generated? Typically, such laws are based on an observation of some regularity (e.g., a correlation) and then use clever experimental designs to manipulate individual factors or conditions as specifically as possible (i.e., without changing other factors).

In the following, I will describe examples from the neurosciences (as one example of a “physical science”), from cognitive neuroscience research (that attempts to bridge psychology and neuroscience), and from psychoanalysis that all refer to representations. From these descriptions, it will become apparent that such laws are always preliminary suggestions for general relationships built on an incomplete knowledge of boundary conditions. *Such weak and preliminary laws are all we have to explain the world, though, and they do not differ systematically between neuroscience, psychology, and psychoanalysis*.

Let's start with neuroscience. To take an example for which last year's Noble prize in Medicine or Physiology was awarded, the activity of “place cells” in the hippocampus was found to correlate with the spatial location of an animal (O'Keefe and Dostrovsky, 1971). Subsequent research identified the boundary conditions of place cell activity at an impressive degree of detail. Now, the brain (similar to the mind) is a complex inter-connected system, and manipulating one factor often influences other processes as well. Thus, the art of experimental research consists in specifically manipulating only one factor at a time, and leaving all others equal. Moreover, knowledge of the brain (and of the mind) is incomplete and fragmentary, and therefore one can only speculate about the possible influences that specific brain processes exert on others—and on all the boundary conditions that need to be met so that place cells can do their job properly. However, this does not mean that no relevant lawful relationships can be found—one is saying something relevantly general and lawful in saying that hippocampal place cells represent spatial locations in various animals (including bats, in whom they are three-dimensional), and much about their properties, development, and functional role has been found out in the last decades.

The situation is quite similar in the cognitive neurosciences, in which psychological processes or properties are explained by brain mechanisms. For example, the capacity of the human working memory store—a psychological concept—is generally limited to something between 3 and 7 items (depending on the metric used). According to one influential theory, this capacity corresponds to the frequency ratio of fast and slow brain oscillations (Lisman and Idiart, 1995). This theory suggests that each cycle of a fast oscillation represents one single item and occurs super-imposed on a specific phase range of a slow oscillation cycle. The more fast cycles fit onto one slow cycle, the more items someone can keep in working memory. Now, this theory is far from being proven, and may actually turn out to be incorrect. However, this is not because the list of boundary conditions for this relationship still needs to be clearly defined, which will very likely never be the case. In general, with the increasing application of multivariate pattern classification analyses, neural representations of specific experiences and concepts can be traced in the brain. Again, these representations are loosely coupled to their content and they are holistic, but that exactly corresponds to the properties of propositional states.

Finally, what about psychoanalysis? The concept of repression is central to psychoanalytic theory and practice and has therefore been a matter of intense investigations, both in the clinical interaction between psychoanalyst and patient and in “extraclinical” experimentation. We have recently adopted free association paradigms similar to those initially conducted by Jung (1918) and investigated memory to either standardized or individualized (autobiographic) conflict sentences (which were derived using “Operationalized Psychodynamic Diagnostics”, OPD-Task-Force, 2008). Consistent with psychodynamic theory, free associations to conflict sentences were accompanied by indirect measures of resistance (longer reaction times) and autonomic arousal (higher skin conductance responses) and were less likely to be remembered afterwards, potentially indicating repression (Kehyayan et al., 2013; Schmeing et al., 2013). Even though we are far from a complete understanding of all factors influencing these relationships, we believe that our findings operationalize and explain at least some important aspects of repression, and could become clinically relevant to dissociate the various (meta-)psychological and brain processes underlying repression. Notably, “reasons” for repression in the context of our paradigms (i.e., the relationship of cue words or sentences to intrapsychical conflicts) would explain behavior strictly analogous to causes in neuroscientific approaches to representations.

Now, are these mundane examples really examples of “scientific laws” in the sense that Galgut is referring to? After all, they constitute only tentative and relatively specific relationship between certain events and properties, will potentially be falsified and very likely be replaced one day by better, more encompassing relationships. They are certainly less strict than laws in the physical sciences which do not relate to representations. However, when representations come into play, they are all we have to explain neuroscientific, psychological, and (as a special case of psychological) psychoanalytic observations. With regard to psychoanalysis, such relationships provide the cornerstone of psychoanalytic metapsychology. In fact, metapsychological theories are typically derived from empirical observations: In Freud's case, from 19th century physiology, such as Helmholtz's laws, which set the basis for the “constancy principle”; in modern psychoanalysis, from various sources including the affective neurosciences, which may be about to radically change the way we think about the mental apparatus (Solms, 2013). The fact that existing psychoanalytic concepts are already intimately intermingled with concepts from the empirical sciences provides clear practical evidence against the purported “inconsistency” between these areas of science (see Flores Mosri et al., 2015).

## Conflict of interest statement

The author declares that the research was conducted in the absence of any commercial or financial relationships that could be construed as a potential conflict of interest.
